# An mHealth App to Support Fertility Patients Navigating the World of Infertility (Infotility): Development and Usability Study

**DOI:** 10.2196/28136

**Published:** 2021-10-12

**Authors:** Katya Kruglova, Siobhan Bernadette Laura O'Connell, Shrinkhala Dawadi, Eden Noah Gelgoot, Skye A Miner, Stephanie Robins, Joy Schinazi, Phyllis Zelkowitz

**Affiliations:** 1 Lady Davis Institute for Medical Research, Jewish General Hospital Montreal, QC Canada; 2 Department of Psychiatry, Jewish General Hospital Montreal, QC Canada; 3 Department of Psychiatry, McGill University Montreal, QC Canada

**Keywords:** mHealth app, mHealth development process, infertility, intervention design, mobile phone

## Abstract

**Background:**

The experience of infertility and its treatment engenders considerable stress and is often described as an emotional rollercoaster. A mobile health (mHealth) app may be a novel solution to address the psychoeducational and psychosocial support needs of fertility patients because of its potential to reduce stress and increase patient empowerment. There are a few fertility-related apps that provide information and support to both men and women undergoing fertility treatment; however, none have documented their development and evaluation process.

**Objective:**

This study aims to describe the development and evaluation process of a bilingual mHealth app, *Infotility*, designed to meet the psychoeducational and psychosocial support needs of men and women undergoing fertility treatment.

**Methods:**

To develop the *Infotility* app, we adhered to the Medical Research Council guidelines for the development and evaluation of complex interventions. First, we conducted literature reviews and needs assessment surveys of fertility patients and health care providers who informed the content and design of the app. Second, we tested the intervention with a small group of end users who provided feedback on the design and appropriateness of the app’s content. Third, we evaluated the uptake and usability of the app using a pre-post study design. Finally, we updated the app’s content based on participants’ feedback and searched for partners to disseminate the app to the broader public.

**Results:**

This study is the first to describe the development and evaluation process of an mHealth app for men and women undergoing fertility treatment. The app met its goal in providing fertility patients with a clinician-approved, portable resource for reliable information about medical and psychosocial aspects of infertility and its treatments and a confidential peer support forum monitored by trained peer supporters. Participants rated the engagement, functionality, information, and esthetics of the app positively, with an overall app quality mean score of 3.75 (SD 0.53) and a star rating of 3.43 (SD 0.75), with a total possible score and star rating of 5.00.

**Conclusions:**

By documenting the systematic development and evaluation of the mHealth app for men and women undergoing fertility treatment, this paper can facilitate the replication of the study intervention and the development of similar mHealth apps.

## Introduction

### The Negative Psychological Consequences of Infertility

Infertility is defined as the inability to achieve pregnancy after 12 months of unprotected sexual intercourse or the inability to reproduce either as an individual or as a couple [1]. Estimates suggest that 11%-16% of Canadians experience infertility in their lifetimes [2]. Infertility is a challenging experience, with infertile individuals demonstrating higher levels of stress, anxiety, depressive symptomatology, and stigma compared with their fertile counterparts [3-5]. Although infertile women tend to feel more stigmatized by infertility and report higher levels of depression and self-blame compared with infertile men [4], men do experience physical and emotional stress, particularly after treatment failure [6]. However, even in cases of male factor infertility, the focus of treatment is usually on the woman’s body, which may make men feel excluded from the fertility treatment process and suspend their own emotional needs to meet the support needs of their female partners [7]. Fertility patients may also experience stress because of the physically arduous, costly, and time-consuming nature of treatment [3]. Although infertility is stressful, many patients do not present with clinical diagnoses of psychological disorders and often do not seek formal mental health services [8-10]. Fertility patients may benefit from alternative options for support such as psychoeducational materials, practical information about treatment and test procedures, and peer support.

Reviews of psychosocial interventions for fertility patients indicate that they are effective at reducing depressive symptomatology, anxiety, and stress associated with infertility [11-13]. Research suggests that patients desire more information from their health care providers about the emotional and psychological aspects of infertility and about treatment and test procedures [14]. Patients also demonstrate an unmet need for information about accessing psychological support services [15,16] and express interest in online peer support [17]. In addition to the clinical setting, fertility patients often search for web-based health information [18]. However, existing web-based resources for fertility patients often do not meet the standards of readability and accuracy, nor do they contain information about male infertility [19].

### Mobile Health Interventions

Mobile health (mHealth) is the provision of health services and information using a mobile device, such as a smartphone [20,21]. Generally, mHealth interventions have a positive effect on users and can help patients with treatment adherence and symptom monitoring [20,22]. Those interventions that include psychoeducation, online peer support, and cognitive-behavioral or mindfulness-based therapies can improve clinical outcomes among users with a variety of health conditions [23-25]. Evidence suggests that mHealth can foster behavioral changes [20,26]. mHealth apps also have the potential to increase patient involvement and feelings of control over the treatment process [27] and provide more personalized care to users. The interactive and multimedia nature of mobile devices also allows for innovation in the presentation of information. This is an important aspect of health-related interventions; visual appeal is related to patient trust, perceived ease of use, and increased understanding [28].

Almost 70% of Canadian adults aged 18 years and older report owning a smartphone, and the rates of internet use and smartphone ownership continue to increase worldwide [29]. Therefore, mHealth may be an effective way to target patient populations who traditionally experience barriers to accessing formal health care services, such as men, immigrants, ethnic minorities and people with stigmatized illnesses [30-33]. Given its potential to reduce stress, increase patient empowerment, and provide user-friendly information to a broad range of the population, an mHealth app may be a novel solution to address fertility patients’ psychoeducational and psychosocial support needs.

### Documenting the mHealth App Development Process

To the best of our knowledge, there are a few fertility-related apps that provide information and support to both men and women undergoing fertility treatment, but none have documented their development and evaluation process. Improved documentation of app development will allow future researchers to develop similar mHealth apps. It will also help in knowledge transfer, which may be especially useful for the more innovative aspects of mHealth, such as design principles, the presentation of information, and technical features.

Accordingly, this study describes the development process for *Infotility*, a bilingual mHealth app designed to meet the psychoeducational needs of both men and women undergoing fertility treatment. *Infotility* contains information about treatments and test procedures, financial and legal aspects of fertility treatment, fertility health and risks to fertility, mental health and wellness, and a confidential forum monitored by peer supporters. In developing this intervention, we adhered to the Medical Research Council (MRC) guidelines for the development and evaluation of complex interventions [34]. As recommended, we used both quantitative (surveys) and qualitative (focus groups or interviews) methods during the development and evaluation of the intervention. We outline the development of the *Infotility* app based on the following recommended steps from the MRC guidelines:

1. *Development of the intervention*: literature review of existing interventions, needs assessments to determine stakeholders’ perspectives on the content of the intervention
2. *Feasibility and piloting*: testing procedures, estimating recruitment and retention, and determining sample size
3. *Evaluation of the intervention*: pre-post study exploring fertility patients’ experiences using the app
4. *Implementation*: dissemination, surveillance and monitoring, and long-term follow-up

## Methods

### Development of Infotility

#### Existing Sources of Web-Based Information and Support for Fertility Patients

To determine whether there were existing mHealth apps that provided fertility information to both men and women, we reviewed the Apple iTunes and Google Play stores between October and December 2016, using the keywords *fertility/fertility* and *infertility/infertilité*. The search revealed numerous menstrual tracking apps and 2 sperm home testing kit apps, *Trak:* Sperm Health and Fertility (Sandstone Diagnostics) and *YO* Sperm Analyzer (Medical Electronic Systems). In a further search of the gray literature, we found that one menstrual cycle app, *Glow*, was developing a component for men that would include fertility-related information, but this was not yet available for review [35]. Our research team did not find any English or French language mHealth apps that addressed both male and female reproductive health concerns.

As part of a project to assess fertility-related information geared specifically for men, our research team performed an analysis of fertility-related health information found on the websites of fertility clinics and major North American organizations [19]. We assessed the quality and readability of information presented on the websites of 28 Canadian fertility clinics and 13 North American organizations such as the Mayo Clinic and RESOLVE. Quality ratings were assessed using the DISCERN instrument [36]. The quality of information found on the North American organizations’ websites was deemed *good*, and the quality of information on the fertility clinic websites received a grade of *fair*. Furthermore, the North American organizations’ websites required an average reading level of 12.9 years of education, and the fertility clinic websites required an average level of 14.3 years, far above the grade 5-8 reading level recommended for health information material [37,38]. The results of this analysis served as a proof of concept for *Infotility*, indicating a lack of web-based sources of high-quality information about reproductive health that might be easily understood by the general population.

In parallel, our research team conducted an internet search to review how infertility blogs and forums offered social support, how they were managed, and what information and features were available to users. Some of the reviewed web-based platforms offering peer support were RESOLVE, Reddit, Association Infertilité Québec, and Fertility Matters Canada. The search showed that infertility forums posted public (openly visible) and private (member log-in required) conversations on a variety of subjects surrounding the experiences of infertility. Moderators or administrators were available to provide feedback on some conversations (or *threads*) between users. Users were required to make an anonymizing username and icon (ie, one that did not identify them), and many forums had rules related to posting, such as the prohibition of using derogatory terms or naming specific professional or clinics. Most sites also offered freely available informational content, such as their own description of fertility or pregnancy, explanations of terms or procedures used during infertility treatment, and tools for tracking ovulation. Fertility forums existed in French or English, and some, such as Reddit [39], provided content geared specifically to men.

#### Needs Assessment Surveys

##### Overview

Contextual inquiry involves assessing the needs and preferences of end users (the population that will use the mHealth app) and key stakeholders (those involved in the creation, evaluation, and distribution of the mHealth app). For *Infotility*, end users are men and women undergoing fertility treatment and the key stakeholders are patient advocates and health care providers (HCPs) in the field of reproductive health. Identifying their needs and values (and later, evaluating whether the needs and values were met) allows for improved buy-in and increased potential for app distribution. To execute a thorough contextual inquiry and use a patient-centered approach to app development, our research team undertook needs assessment surveys of fertility patients and HCPs. All survey questions were designed by our research team, including expert clinicians in the field of reproductive health. To our knowledge, this is the first study to document such a process for a fertility-related app, although mHealth interventions for other conditions have used techniques such as interviews, expert panel consultation, and focus groups to better understand their target populations [40,41].

##### Needs Assessment Surveys of Fertility Patients’ Use of and Desire for mHealth Apps

A diverse sample of fertility patients was recruited from fertility clinics in Montreal and Toronto to complete the needs assessment survey. A total of 659 patients completed a web-based survey asking them about their experiences of, and preferences for, fertility-related information disseminated through clinical, web-based, and mobile modalities. The sociodemographic characteristics of the survey sample are given in Multimedia Appendix 1. Dawadi et al [15] provided a more detailed description of methods and measures. The results of the needs assessment survey showed that most participants had searched the internet for fertility-related information, underscoring the importance of web-based health resources in addressing the informational needs of fertility patients [42]. Moreover, most participants did not report using a fertility mobile app, but the majority were interested in using one [43]. This finding served as another proof of concept for *Infotility*, as the discrepancy between use of and interest in using a fertility app suggested a lack of publicly available, good-quality web-based resources for fertility patients.

Participants were also asked about their preferred fertility app features. The five most endorsed features were as follows: being easy to understand, including a glossary of medical terms, providing information on fertility health care coverage, providing information on reproductive health, and being free of charge [43]. These findings informed our choice of content topics and features of *Infotility*.

The needs assessment survey also examined the fertility patients’ interest in online peer support and their preferences for various features of an online peer support forum [17]. The majority of the participants expressed interest in using online peer support and endorsed a monitored peer support forum that is accessible on a mobile device, allows participants to connect with peers, contains links to external resources, and is monitored by a health professional. These results affirmed our decision to include a peer support forum in the *Infotility* app and informed the design of the forum.

##### Needs Assessment Surveys of Health Care Providers

To obtain the perspectives of those who have frequent contact with fertility patients in a variety of capacities, our team conducted a needs assessment survey of fertility HCPs, including physicians, nurses, mental health and wellness professionals, and administrative staff. Eligible participants were recruited at 6 sites in Montreal and Toronto, and a total of 127 participants completed a web-based survey. The survey asked fertility HCPs about features they believed a fertility mobile app should include and the types of information that fertility patients typically requested.

The majority of HCPs thought that patients would be interested in using a high-quality mobile app that provides fertility-related information and support. With respect to the app features, the ones that were most highly endorsed by HCPs included being easy to understand, providing information that promotes reproductive health, containing a glossary of medical terms, and offering links to stress reduction tools [44]. Most of the features endorsed by HCPs were also identified by the surveyed fertility patients as most desirable in a mobile fertility app.

The survey also showed that HCPs most frequently provided patients with information on tests and procedures, medications, and explanations of conditions [45]. Comparing these findings with the results of the needs assessment survey of fertility patients demonstrated potential discrepancies between the types and amount of information provided by HCPs and those that fertility patients would have liked to receive. For example, many patients wanted to receive information on insurance and regulations but were less likely to obtain this information from HCPs. On the basis of these findings, our team decided to include detailed information on fertility laws, regulations, and health care coverage in the *Infotility* app.

#### Literature Review, Content Development, and Expert Input

To write the content for the *Infotility* app, our research team conducted a literature review on the topics related to infertility by searching web-based scholarly databases (eg, PubMed, PsycINFO, and Medline) for scientific literature published from 2000 to 2017. We also consulted gray literature, such as articles published in popular news media (eg, The New York Times) to capture personal experiences of fertility patients.

On the basis of the literature review as well as the responses from the needs assessment surveys, our team developed a range of content categories for the *Infotility* app. For each content topic, we created brief summaries of the scholarly and gray literature and collated this information into reports. These reports were then sent to members of the team and advisory committee who specialized in the content area and fertility patients for feedback. For example, a section on the causes of male factor infertility was sent to a urologist, whereas a section on the psychosocial aspects of infertility was sent to a clinical psychologist specializing in fertility counseling. These specialists ensured the clinical relevance and accuracy of the content. The summaries, once approved, formed the basis for the app content, which was edited and presented in clear and accessible language.

The final approved content for the *Infotility* app included a variety of informational topics such as content related to reproductive health, the psychosocial challenges of infertility, and the legal and financial aspects of fertility treatment (a detailed list of content topics are included in Textbox 1).

As the app was targeted to both female and male users, our research team made a concerted effort to identify content that expressed the experiences of both men and women undergoing fertility treatment. We did so by tailoring the language and information of certain content sections (eg, sections on nutrition and exercise) to be different for men and women.

**Content categories and articles of the Infotility app.**

**How to get pregnant**

- Reproduction 101
- All about eggs and ovulation
- Tracking your ovulation
- Frequently asked questions about getting pregnant

**Causes and diagnoses**

- Female factor infertility causes
- Risks to female fertility
- Masturbation
- Other ways to provide a sperm sample
- Causes of male infertility
- Risks to male fertility
- Am I at risk for other health problems?

**Treatment options**

- Ovulation induction
- Intrauterine insemination
- In vitro fertilization
- Sperm, embryo, or egg donation

**Using a donor or surrogate**

- When to consider using donor eggs, sperm, embryos, or surrogate
- Preparing for donation
- Pros and cons of donation
- Challenges of egg, sperm, and embryo donation
- Medical steps and additional information

**Genetic testing**

- How does genetic testing work?
- What are the tests?

**Multiple pregnancy losses**

- Causes and treatments of multiple pregnancy losses

**Stopping treatment**

- Why do people stop treatment?
- If you decide to stop treatment

**Fertility laws and health care coverage**

- Assisted Human Reproduction Act
- What expenses are covered?
- Surrogacy and gamete donation laws
- On embryos as property and human life

**Your relationships**

- Keeping your couple healthy
- How do I talk about infertility?

**Physical well-being**

- Exercise and fertility
- Nutrition for fertility
- Environmental risks to fertility

**Mental well-being**

- Taking care of your body and mind
- Dealing with pregnancy loss

**Working with your health care team**

- Choosing the clinic that’s right for you
- Preparing for medical appointments
- Questions you may want to ask your doctor
- Getting a second opinion: When and why?

In addition, our research team developed a glossary of key fertility-related terms using the *International Committee for Monitoring Assisted Reproductive Technology and the World Health Organization Revised Glossary on Assisted Reproductive Technology Terminology* as a reference [46]. We then added or removed certain terms based on their presence in the *Infotility* app content and edited the glossary to ensure its readability.

To serve the bilingual population of Montreal, Quebec, the app had to be available in both English and French. Therefore, once finalized, the written content was translated into French. The reading level of the content was assessed to ensure acceptability and appropriateness for the target audience. The English content was assessed using the Flesh Kincaid grade level [47], and the French content was evaluated using a web-based tool specifically designed to assess French written information using the Gunning Fog index [48,49]. Both measures are valid and reliable indexes commonly used to assess the grade level of educational knowledge required to understand written information. The measures indicated that the English content was written at an eighth-tenth grade reading level, whereas the French content was at the 12th grade level. This is slightly above the common guidelines recommending that health information material be written at a reading level of grade 5-8 [37,38]. However, previous literature has found that people seeking fertility treatment have, on average, higher education levels than other patient populations [50,51], and our needs assessment survey found that the majority of fertility patients in our sample had a university degree or higher [42]. Taking this into consideration, the reading level of the *Infotility* app is consistent with more flexible recommendations that content be developed at a reading level 1 to 3 grades lower than the mean education level of the target population [52].

In addition to providing information on the psychosocial and medical aspects of infertility in both English and French, the *Infotility* app integrated an online peer support forum *Connect* that allowed users to confidentially post about their experiences, communicate with each other via discussion posts, and ask private questions of a peer supporter. Screenshots of the peer support forum are provided in Multimedia Appendix 2. Peer supporters were current or former fertility patients who completed a training program developed by our research team. Training involved three components: (1) reviewing a peer support manual approved by an expert on peer support manuals [16], (2) watching a training webinar containing practice questions and a discussion of ideal responses, and (3) responding to hypothetical discussion posts followed by feedback from the research team. The peer support manual explained the role of a peer supporter [53], outlined strategies for providing web-based support to participants, and provided basic information about infertility and definitions of common medical terms. Our research team recruited and trained peer supporters to communicate with users via the forum and through private messages. Each peer supporter was asked to monitor the forum between 2 and 4 hours per week. The forum was also monitored by members of the research team to ensure that there were no posts that promoted specific products or clinics, gave medical advice to another participant, or indicated that a participant was distressed or considering self-harm. The team members also monitored the responses provided by peer supporters and were available to answer their questions. A study by Grunberg et al [53] provided a full description of methods.

#### App Design and Operationalization

To pick a name for the app and the peer support forum, members of the research team were polled, and the suggested names were voted on. Ultimately, the name *Infotility* was chosen for the app and the name *Connect* was chosen for the forum as both are short, unique, and easy to remember. Moreover, *Infotility* reflected the main purpose of the app, that is, providing information to fertility patients, and *Connect* embodied the spirit of the forum—connecting people via the internet. As the app had to be bilingual, these names were also chosen because they had suitable French equivalents—*Infotilité* and *Connecte.*

**Figure 1:**
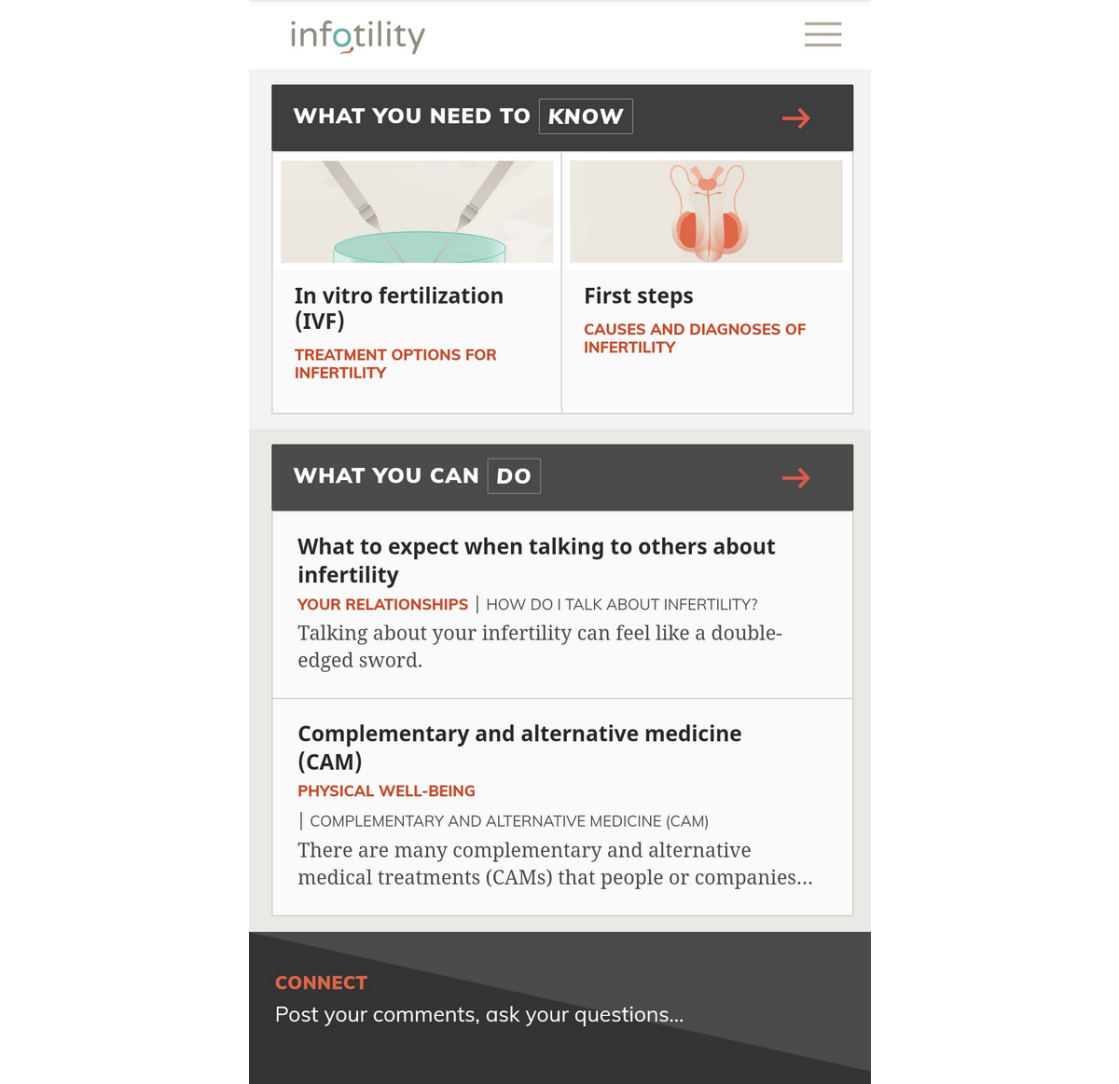
Dashboard of the *Infotility* app.

**Figure 2:**
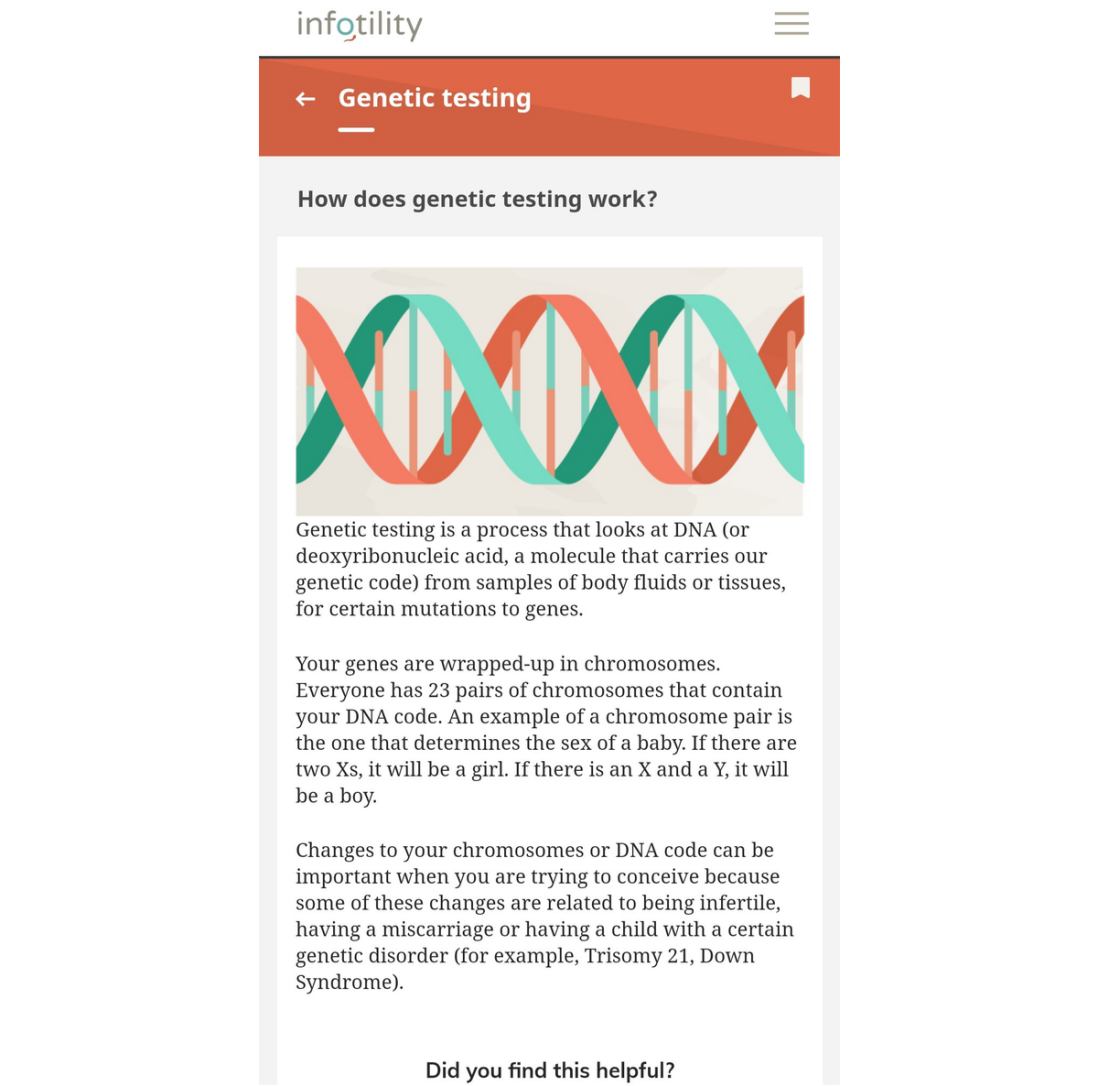
The article on genetic testing.

To design a user-friendly mHealth app, members of our research team participated in a 2-day workshop organized by an independent app design company. During this workshop, we completed a number of design activities, including persona development (representation of a typical user), brand conceptualization, journey mapping of the user, and the creation of hypothetical wireframes (screen blueprints). We then supplied the ideas generated at the workshop to a different app design company with which we collaborated in the development of the app’s user experience (overall experience a user has with the app), user interface (how the app interface functions and how the user interacts with it), and information architecture (how the app’s content is structured). Figures 1 and 2 show examples of the interface and architecture of the app. The process of designing *Infotility* was informed by the principles of user-centered design, which focuses on the needs of the user at all phases of development [54]. With the understanding that the amount of information available to fertility patients can be overwhelming, we ensured that app users were able to choose the information they wished to have presented to them first. The information was further divided into digestible *chunks* and was tied together with an appealing theme to keep the app cohesive. The major organizing sections of the app were *What you need to know*, focusing on informational aspects of going through infertility and its treatment, and *What you can do*, focusing on actionable items, such as exercising for fertility.

Our research team also worked with the app design company to create a theme and a mascot unique to *Infotility*. For the mascot, we chose a sperm whale. Once a color scheme and font were approved, the app design company produced graphics that either complemented or replaced the text in accordance with the guidance provided by the research team, which often included the whale mascot in different scenarios. Examples of the *Infotility* mascot and graphics are given in Multimedia Appendix 3. The intention of these graphics was to break up long blocks of text into smaller paragraphs to make the information easier to read and more appropriate for viewing on a mobile screen. In addition, although men and women had to use different log-in credentials to access the app because certain topics were sex-specific (eg, tips on how to provide a sperm sample for men), we created a feature called the *flip side*, which allowed users to view the opposite sex’s app content. When a user clicked the *flip side* function, the app pages were animated to *flip* to see the opposite sex’s information.

### Feasibility and Piloting

An interactive prototype of the *Infotility* app was made available on a web-based platform. This prototype allowed us to discuss the flow and organization of information. Once the organization was approved, the app company consulted 2 male end users and 3 female end users who had undergone or were undergoing fertility treatment. These users provided feedback on the usability of *Infotility* and the design and appropriateness of the content. On the basis of this feedback, our research team adjusted the design, language, and tone of the app.

The sample size for the evaluation of the intervention was informed by guidelines for pilot studies of web-based interventions, which recommend at least 20 users [55]. To account for attrition and explore differences between men and women, we aimed to have at least 50 men and 50 women successfully finish the study by using the *Infotility* app for 8 weeks and completing questionnaires both before and after using the app.

### Evaluation of Infotility

The primary goal of the study is to create an app that is user friendly, provides peer support, and contains reliable and easy-to-understand information about all aspects of infertility and its treatment. In the final steps of the study, we evaluated the uptake and usability of the *Infotility* app using a pre-post study design.

#### Participants and Procedures

Fertility patients were recruited from fertility clinics in Montreal and Toronto from October 2018 to December 2018 to test the *Infotility* app. Eligible participants met the following criteria: they were aged ≥18 years, were in a heterosexual relationship, read English and/or French, had access to the internet, and identified as male or female. In the initial stages of recruitment, we limited our inclusion criteria to only those undergoing in vitro fertilization for the first time. However, based on recommendations from our clinician partners, we expanded the inclusion criteria to recruit any fertility patients at any stage of treatment. There was great interest in the study among patients, and in the interest of being inclusive to all those who wished to participate, we did not limit the number of people who consented to participate in the study, even after our goal of 50 women and 50 men was achieved. This also allowed us to recruit a diverse sample and mitigate participant dropouts. When we felt we had a sufficient number of participants enrolled in the study to account for attrition over the 8-week study period, we stopped recruitment and determined April 30, 2019, as the study end date.

A total of 969 people (336/969, 34.6% men and 633/969, 65.3% women) were approached by recruiters. Furthermore, 661 (220/661, 33.2% men and 441/661, 66.7% women) agreed to be screened for eligibility, 505 (164/505, 32.4% men and 341/505, 67.5% women) were eligible to participate, and 387 (124/387, 32.0% men and 263/387, 67.9% women) consented to participate in the study. Before being given access to the *Infotility* app, participants were asked to complete a number of intake questionnaires measuring demographic characteristics, fertility treatments and diagnoses, fertility-related quality of life, psychological distress, and lifestyle habits, which took approximately 30 minutes to complete. Of the 387 who consented, 26% (65/250) men and 74% (185/250) women completed the intake questionnaires and visited the *Infotility* app at least once. Participants’ sociodemographic characteristics are given in Multimedia Appendix 4. Participants were given access to *Infotility* for 8 weeks and could use the app as much or as little as they liked during the study period. Once using the app, participants could choose whether to access the peer support forum. After 8 weeks of using *Infotility*, of the 250 participants who used the app, 22.1% (38/172) men and 77.9% (134/172) women completed the follow-up questionnaires measuring participants’ evaluations of and experiences using the app, in addition to the same measures administered at intake. Participants who completed the study were sent a Can $25 (US $32) gift card. For the purposes of this paper, data evaluating participants’ experiences using the *Infotility* app, including the peer support forum *Connect*, are presented.

#### Measures

Google Analytics was used to collect data on how participants used the *Infotility* app during the 8-week study period. We gathered several key performance indicators to assess the frequency and patterns of app use, including the number of page views and total time spent on the app.

Participants were sent the user version of the Mobile Application Rating Scale (uMARS) to evaluate their satisfaction with the app and whether their needs and preferences were met [40]. The uMARS includes four subscales asking users to rate the app’s quality of engagement, functionality, esthetics, and information [56]. Each item is measured on a Likert scale from 1-5, with higher scores representing higher quality ratings. The total scores for each subscale were generated by summing the individual items of the subscale and dividing it by the number of items in the subscale. The total app quality mean score was calculated by adding the scores from the four subscales together and dividing by four. There are four additional items of the uMARS that can be averaged to give an app *subjective* quality mean score. The research team chose the uMARS because it is a valid (α=.90) and reliable (test-retest reliability after 3 months = 0.63-0.85) measure of user satisfaction with mHealth apps and tailored toward the experiences of patients rather than professionals who work in technology or health care [56]. Three additional open-ended questions were developed by our research team to be administered after completing the uMARS, asking participants to describe (1) any topics or features that were not included in the app that they would have liked to be included, (2) what they liked best about the app, and (3) what they liked least about the app.

The Peer Support Evaluation Inventory (PSEI) was used to evaluate user satisfaction with the peer support forum. The PSEI was developed by our research team and adapted from a measure by Dennis [57]. The PSEI includes four subscales measuring supportive interactions, relationship qualities, perceived benefits, and satisfaction, with support received from the peer support forum. Each item is measured on a Likert scale from 1-4, with higher scores representing higher levels of user satisfaction with the peer support forum. The total scores for each subscale were generated by summing the individual items of the subscale and dividing by the number of items in the subscale.

#### Research Design and Analytic Strategy

Quantitative analyses were used to evaluate whether we achieved our goals of making the *Infotility* app useful and accessible to a sample of participants undergoing fertility treatment. Descriptive statistics of the uMARS and PSEI subscales present participants’ overall ratings of the *Infotility* app and peer support forum. Bivariate analyses were used to determine whether there were any associations between the uMARS scores and app use. Qualitative responses to the three open-ended questions were read by 2 researchers, and common themes about the strengths and weaknesses of the app were identified and presented as supplementary data to the quantitative findings.

## Results

### Quantitative Findings

On average, participants visited approximately 34 pages and spent 22 minutes on the *Infotility* app. Participants rated the engagement, functionality, information, and esthetics of the app positively, with an overall app quality mean score of 3.75 (SD 0.53) and a star rating of 3.43 (SD 0.75), with a total possible score and star rating of 5.0. When asked whether they would recommend the app to other people who might benefit from it, 49.7% (94/189) responded “definitely” or “there were many people I would recommend this app to.” When asked how many times they would use the app in the next 12 months, 5.7% (11/191) said they would use it more than 50 times, 25.6% (49/191) said 10 to 50 times, 42.9% (82/191) said 3 to 10 times, 19.3% (37/191) said 1 to 2 times, and 6.2% (12/191) said they would not use it. When asked if they would pay for the app, approximately 50.2% (95/189) said “Definitely not,” and 2.1% (4/189) said “Definitely yes.” A subsample of 106 *Infotility* users used the *Connect* forum. On average, *Connect* users rated the supportive interactions of peer supporters 2.91/4 (SD 0.84), the relationship qualities 2.95/4 (SD 0.49), their perceived benefits 2.92/4 (SD 0.74), and their satisfaction with support received 2.89/4 (SD 0.82; Table 1).

**Table 1:** App use and user ratings of the Infotility app and the Connect peer support forum (N=250).

Characteristics			Value, n (%)		Mean (SD)		Range
**App use (Google Analytics)**							
	Total page views	250 (100)		33.93 (35.14)		1.00-270.00	
	Time spent on app (minutes)	250 (100)		22.07 (29.98)		0.00-193.18	
**uMARS^a^**							
	Total app quality	167 (66.8)		3.76 (0.53)		2.40-5.00	
	Engagement subscale	186 (74.4)		3.33 (0.64)		1.40-5.00	
	Functionality subscale	188 (75.5)		3.96 (0.65)		1.75-5.00	
	Esthetics subscale	190 (76)		3.73 (0.66)		1.67-5.00	
	Information subscale	174 (69.6)		3.97 (0.61)		2.00-5.00	
**Would you recommend this app to people who might benefit from it? (n=189)**							
	Not at all	9 (4.7)		N/A^b^		N/A	
	Very few people	23 (12.1)		N/A		N/A	
	Maybe	63 (33.3)		N/A		N/A	
	There are many people I would recommend this app to	51 (26.9)		N/A		N/A	
	Definitely	43 (22.7)		N/A		N/A	
**How many times do you think you would use this app in the next 12 months if it were relevant to you? (n=** **191)**							
	None	12 (6.2)		N/A		N/A	
	1-2	37 (19.3)		N/A		N/A	
	3-10	82 (42.9)		N/A		N/A	
	10-50	49 (25.6)		N/A		N/A	
	>50	11 (5.7)		N/A		N/A	
**Would you pay for this app? (n=** **189)**							
	Definitely not	95 (50.2)		N/A		N/A	
	2	36 (19.0)		N/A		N/A	
	3	37 (19.5)		N/A		N/A	
	4	17 (8.9)		N/A		N/A	
	Definitely yes	4 (2.1)		N/A		N/A	
**What is your overall (star) rating of the app? (n=** **186)**							
	1	2 (1)		N/A		N/A	
	2	12 (6.4)		N/A		N/A	
	3	87 (46.7)		N/A		N/A	
	4	74 (39.7)		N/A		N/A	
	5	11 (5.9)		N/A		N/A	
**PSEI^c^**							
	Supportive Interactions subscale	98		2.91 (0.84)		1.00-4.00	
	Relationship Qualities subscale	91		2.95 (0.49)		2.00-4.00	
	Perceived Benefits subscale	90		2.92 (0.74)		1.00-4.00	
	Satisfaction With Support Received subscale	86		2.89 (0.82)		1.00-4.00	

^a^uMARS: user version of the Mobile App Rating Scale.

^b^N/A: not applicable

^c^PSEI: Peer Support Evaluation Inventory.

Table 2 presents the correlations between the indicators of app use and the uMARS ratings of the app. Participants’ scores on the functionality subscale were positively correlated with the number of page views (*r*=0.150; *P*=.04 for n=182) and the amount of time spent on the app (*r*=0.185; *P*=.01 for n=182). In addition, scores on the engagement subscale were correlated with the amount of time spent on the app, with a *P* value approaching significance, (*r*=0.142; *P*=.06 for n=180). This means that participants who felt the app functioned well and was easy to learn and navigate visited more pages and spent more time on the app, and participants who felt the app was more engaging spent more time on the app. Participants’ scores on the esthetics and information subscales were not significantly correlated with app engagement.

**Table 2:** Pearson correlations between user ratings and engagement of the Infotility app.

uMARS^a^ subscale	Total page views		Total time spent on the app	
	*r*	*P* value	*r*	*P* value
Engagement subscale	0.046	.54	0.142	.06
Functionality subscale	0.150^b^	.04	0.185^b^	.01
Esthetics subscale	0.096	.19	0.128	.08
Information subscale	0.051	.51	0.081	.30

^a^uMARS: user version of the Mobile App Rating Scale.

^b^Significant at α=.05.

### Qualitative Feedback

Qualitative responses to the three open-ended questions administered at follow-up highlight some important findings about the strengths and limitations of the *Infotility* app. Overall, participants expressed that they appreciated the app: the “information is clear and easy to find” (Participant #03-0046), “it’s a good tool to help and inform about infertility” (Participant #03-0014), and using the app was “more reassuring than googling a question” (Participant #03-0120). Some participants had suggestions for ways to improve the app, such as including more interactive features, communication with medical professionals, and informative videos:

An app usually should have some sort of interactive feature that brings you back, such as a fertility medication tracker, symptoms tracker, etc.

Participant #03-0283

It would be nice if this app was different, in the way that, it was live and interactive with specialists

Participant #03-0248

I think I would be happy to see short videos with the doctors of the clinic giving advices or telling a bit about their experience and statistics in infertility treatment

Participant #03-0052

There was also a desire for more in-depth information that was updated and customized to individual participants and personal testimonies from others:

No newsfeed feature. Update dashboard with new content/information based on my preferences. Why? To draw my attention, make me want to log in more often. I would see the same info each time I open the app. I would only log in if I’m looking for something in particular.

Participant #03-0038

There is a need for medical information that may or may not apply to you. Maybe cases? Personal stories? What happens to other people?

Participant #03-0104

Finally, many participants expressed that the peer support forum was their favorite part of the app and that using it reduced feelings of isolation, helped them manage stress, and provided valuable information. The *Connect* forum was perceived as confidential and safe, and peer supporters helped keep conversations respectful and on track [58].

## Discussion

### Principal Findings

To the best of our knowledge, this is the first study to describe the development and evaluation process of an mHealth app providing information and support to both men and women undergoing fertility treatment. The *Infotility* app was developed by our research team and was informed by extensive literature reviews and needs assessment surveys of fertility patients and HCPs. The content of *Infotility* was reviewed and approved by physicians, nurses, psychologists, and experts in the field of fertility to ensure its clinical relevance and accuracy. *Infotility* was designed by an app design company who worked alongside our team to ensure that the app was user friendly and esthetically pleasing. In addition to providing over 40 articles on the psychosocial and medical aspects of infertility, the *Infotility* app included a confidential peer support forum monitored by trained peer supporters.

In developing the *Infotility* app, our team adhered to the MRC guidelines for the development and evaluation of complex interventions, which provided a structured framework on how to approach the development, piloting, evaluation, and implementation phases of the study. By providing a complete description of all the steps of the app development process, this study can facilitate future replications of the study intervention and the development of similar mHealth apps.

### Development of the Intervention

The needs assessment surveys of fertility patients and HCPs were a crucial step in identifying the informational and psychosocial needs of the end users of *Infotility*. By recruiting a large and sociodemographically diverse sample, we were able to gain insights into fertility patients’ experiences searching the internet for fertility-related information and their preferred features of a fertility app. Furthermore, obtaining the perspectives of fertility HCPs provided valuable insights into the types of information fertility patients most often requested and whether their needs were met.

The results of the needs assessment surveys guided our team throughout the entire development process of *Infotility* with respect to both its content and design. These results were especially helpful when deciding whether to include certain features on the app, which are costly and time consuming to develop. For example, the peer support forum and the detailed medical glossary were features of the *Infotility* app that took much time to develop and design. Despite this, we felt justified in including them on the app and confident in dedicating the time and resources to develop them based on the overwhelming evidence from our needs assessment surveys that these features would be beneficial to fertility patients. Furthermore, needs assessment surveys can provide insights into what you should *not* spend time and resources developing when the target audience informs you that they do not feel the need for certain features or topics. For example, we decided not to include celebrities’ stories of dealing with infertility because they were rated as one of the least desired features by the surveyed fertility patients and HCPs. The results from the needs assessment surveys allowed our team to feel confident and justified throughout the entire app development process.

### Feasibility and Piloting

The second stage of the MRC guidelines involves pilot-testing to determine the feasibility of complex interventions. This study assessed the feasibility of recruitment and retention of male and female participants and evaluated fertility patients’ satisfaction with the *Infotility* app using the uMARS and qualitative interviews.

Recruitment of patients in waiting rooms of fertility clinics proved feasible: of 969 people approached by recruiters, 505 (52.1%; 64/505, 32.4% men and 341/505, 67.5% women) were eligible to participate and 39.9% (387/969; 124/387, 32.0% men and 263/387, 67.9% women) consented to participate in the study. However, recruitment of participants in fertility clinics might have excluded those with lower socioeconomic status who may not be able to afford fertility treatment. Nevertheless, within the limits of recruiting individuals who seek fertility treatment, we were able to obtain a sample that was diverse with respect to participants’ household income (with about 30% below the median Canadian family income), ethnicity, immigrant status, and religion (Multimedia Appendix 4). Recruitment at fertility clinics might also have limited the sample size by excluding men and women who were not seeking fertility treatment but could have, however, benefited from the fertility-related information included in the app. Furthermore, this study only included heterosexual people; future studies should consider including single and nonheterosexual people to explore the experiences and psychoeducational needs of a more diverse population. In addition, the lag time between the needs assessment surveys and the launch of recruitment and data collection could have impacted the feasibility of the app intervention, as informational and support needs of end users may have evolved. Researchers planning to create similar tools should be cognizant of the inherent complexity of the development of an mHealth app and potential unanticipated delays during the process.

It is also worth noting that in line with the previous research regarding the lack of male participants in reproductive research [59], our team experienced difficulties in achieving the recruitment target of men and retaining those who agreed to participate in the study. For example, men were more likely to discontinue at some point throughout the study than women. Future studies should carefully consider the issues of recruitment and engagement of men to ensure that the psychoeducational needs of men with fertility concerns are addressed.

### Evaluation of the Intervention

The evaluation of an mHealth intervention before disseminating it to a larger population or to the general public is necessary to ensure that it will be beneficial to its end users and successful in its proposed objectives. The pre-post study design proved to be effective in capturing fertility patients’ experiences interacting with the *Infotility* app, including the peer support forum *Connect*. In general, participants enjoyed using *Infotility* and rated the informational side of the app and the peer support forum positively. The results showed that those who spent more time on *Infotility* were also those who rated the app higher on functionality (ie, how well the app functions and how easy it is to navigate) and engagement (ie, customization and interactivity). Our findings suggest that when developing mHealth apps, researchers and medical professionals should make the app engaging through interactive features and feedback and implement quality assurance procedures to address any technical issues that, if not resolved, may affect the level of trust among users and lead to user discontinuation [60].

The collection of both qualitative and quantitative data for the pre-post app evaluation provided nuanced and in-depth information about the app users’ experiences using *Infotility* and what they felt the strengths and weaknesses of the app were. In addition, tracking participants’ actual app use data through Google Analytics was highly informative and allowed our team to examine participants’ self-reported data within the context of the pages they accessed on the app.

By tracking participants’ actual app use data through Google Analytics, we were also able to gain insights into the amount of time participants spent on *Infotility* and whether they visited the app multiple times throughout the 8-week study period. Data showed that most participants used the app within the first 2 weeks of the study period, which might help explain the high attrition rate of the study. Although high attrition is common in studies of mHealth interventions [61], our findings suggest that using a study period of less than 8 weeks in future studies may help reduce attrition while providing sufficient information about participants’ app use.

Careful analysis of participants’ app use along with their qualitative and quantitative feedback is critical to improving user experiences of future versions of the mHealth app. For example, future iterations of *Infotility* could be improved by including more interactive features that *bring you back* (eg, informative videos), more in-depth information on certain topics that were not extensively covered in the app, and summaries of current research in accessible language. Implementation of these features will help make future versions of the *Infotility* app more comprehensive and interactive, thereby potentially improving the rates of app engagement and user satisfaction.

### Implementation

Finally, the evaluation of the *Infotility* app through a pre-post study design was necessary to facilitate the fourth stage of the MRC guidelines, which is implementation that includes dissemination, surveillance, and long-term follow-up.

Conducting repeated reviews of scientific literature is necessary to keep the content of *Infotility* accurate and up to date. Upon completion of the study, the entire content of the app was reviewed and edited by our research team based on feedback from the participants and by a knowledge translation consultant with expertise and personal experiences in the fertility field. Changes were made through a content management system, which is a user-friendly and accessible program that allowed our team to edit the app content in real time, without the need to outsource this task to the app company.

As this study is now complete, our team will search for partners that can maintain and disseminate *Infotility* to the broader public. We will identify potential app host partners, such as nonprofit health care organizations and health research institutes, whose strategic plan’s objectives fit the mission of *Infotility*. We will then contact these organizations with a proposal and an estimated budget required to keep *Infotility* up to date, including the costs of literature and policy reviews, summarizing the information in lay terms, content translations and integration, and technical costs associated with domain registry and website hosting. On the basis of the results of the pre-post study, we can confidently conclude that fertility patients in Quebec and Ontario appreciated and enjoyed using the *Infotility* app, which will make the app more attractive to potential partners. Building a long-term partnership will help ensure that the *Infotility* app continues to provide accurate and reliable information to fertility patients.

### Conclusions

Overall, the *Infotility* app succeeded in its goal to provide men and women undergoing fertility treatment with information and support through a user-friendly, credible, and single-source tool. The development and evaluation of *Infotility* highlighted the important aspects of the app creation process, which may be beneficial for researchers and medical professionals who wish to create similar mHealth apps in the future.

## Appendix

Multimedia Appendix 1 Sociodemographic characteristics of fertility patients who responded to the needs assessment survey (N=659).

Multimedia Appendix 2 Screenshots of the peer support forum Connect.

Multimedia Appendix 3 Screenshots of the Infotility mascot and graphics.

Multimedia Appendix 4 Sociodemographic characteristics of study participants (N=250).

## Abbreviations

HCP: health care provider
mHealth: mobile health
MRC: Medical Research Council
PSEI: Peer Support Evaluation Inventory
uMARS: user version of the Mobile Application Rating Scale
