# Supplementary material for: An mHealth App to Support Fertility Patients Navigating the World of Infertility (Infotility): Development and Usability Study
Source: JMIR Form Res. 2021 Oct 12;5(10):e28136. doi: 10.2196/28136 (PMC8548975; doi:10.2196/28136)
Supplement: Multimedia Appendix 1 [file formative_v5i10e28136_app1.pdf]

Socio-demographic characteristics of fertility patients who responded to the needs assessment survey (N=659)

	n	Valid % or *M*(*SD*)(Range)
**Age (years)**	574	36.5(5.48)(22-62)
**Gender**		
Male	289	43.9
Female	370	56.1
**Language**		
English	413	62.7
French	246	37.3
**How many children do you have?**		
None	472	72.2
1 or more	182	27.8
**Sexual orientation**		
Heterosexual	567	96.3
Non-heterosexual	22	3.7
**Current marital status**		
Single	21	3.7
Long-term dating	16	2.8
Living with partner	106	18.5
Married	425	74.0
Separated	4	0.7
Divorced	1	0.2
Widowed	1	0.2
**Total household income (CAD)**		
≤ $20,000	40	7.1
$20,000-39,999	71	12.5
$40,000-59,999	89	15.7
$60,000-79,999	72	12.7
$80,000-99,999	71	12.5
$100,000-119,000	93	16.4
$120,000 and above	130	23.0
**Highest level of education**		
Less than high school	3	0.5
Some high school	10	1.7
High school diploma	36	6.3
CEGEP[a]	101	17.7
Some university	45	7.9

University	233	40.7
Some post-graduate study	22	3.8
Post-graduate study	122	21.3
**Immigrant status**		
Immigrant	274	47.6
Born in Canada	302	52.4
**Ethnicity**		
Aboriginal	6	1.0
Arab	43	7.5
Black	54	9.4
Chinese	25	4.4
Filipino	12	2.1
Japanese	3	0.5
Korean	3	0.5
Latin American	39	6.8
South Asian	36	6.3
Southeast Asian	3	0.5
West Asian	7	1.2
White	289	50.3
Other	54	9.4
**Do you consider yourself a religious person?**		
Yes	291	50.8
No	282	49.2

[a] CEGEP is a two-year college preparatory program or three-year technical program following high school and preceding post-secondary education in Quebec, Canada.
