# Supplementary figures and images for: An mHealth App to Support Fertility Patients Navigating the World of Infertility (Infotility): Development and Usability Study
Source: JMIR Form Res. 2021 Oct 12;5(10):e28136. doi: 10.2196/28136 (PMC8548975; doi:10.2196/28136)

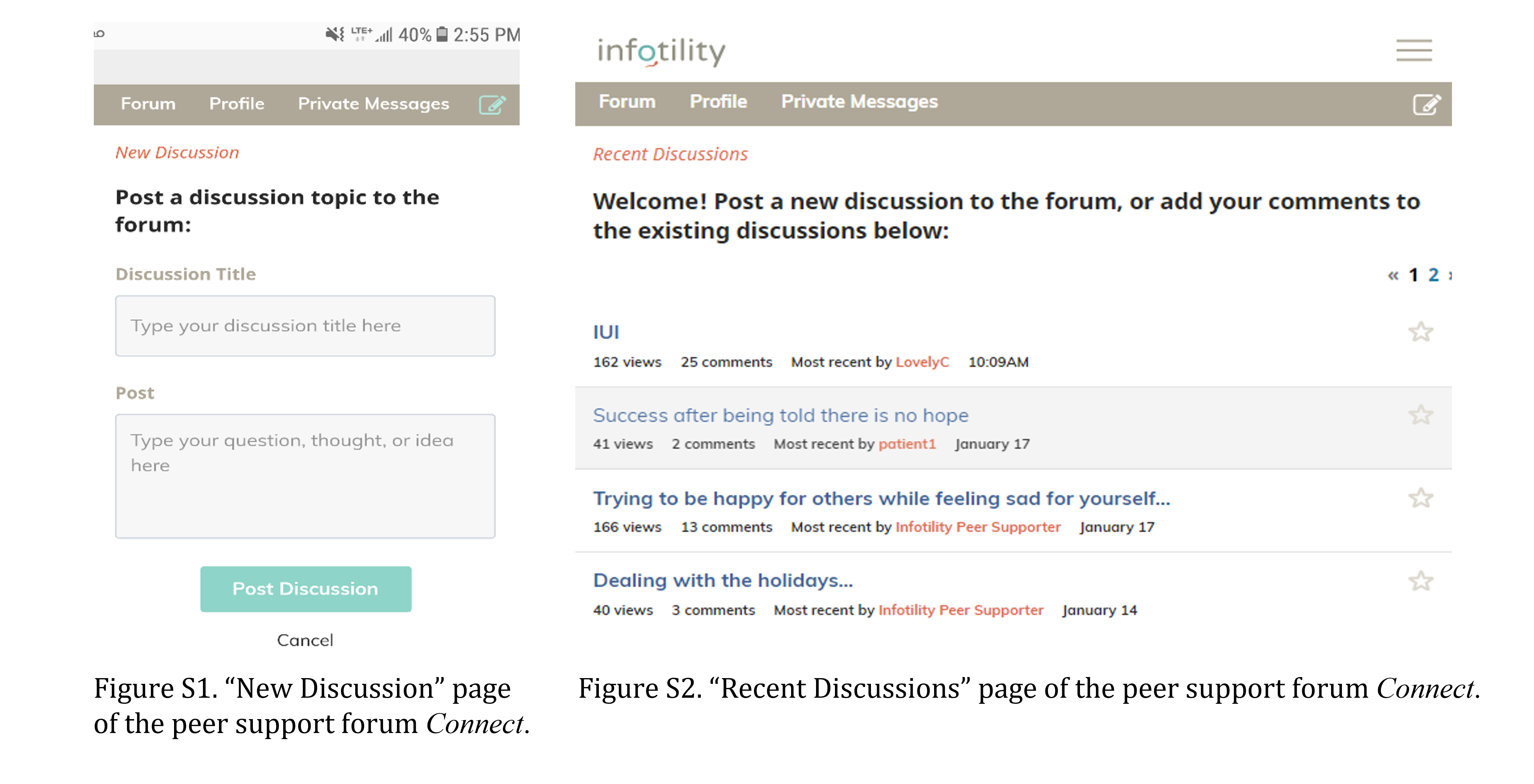

Supplement: Multimedia Appendix 2 [file formative_v5i10e28136_app2.png]

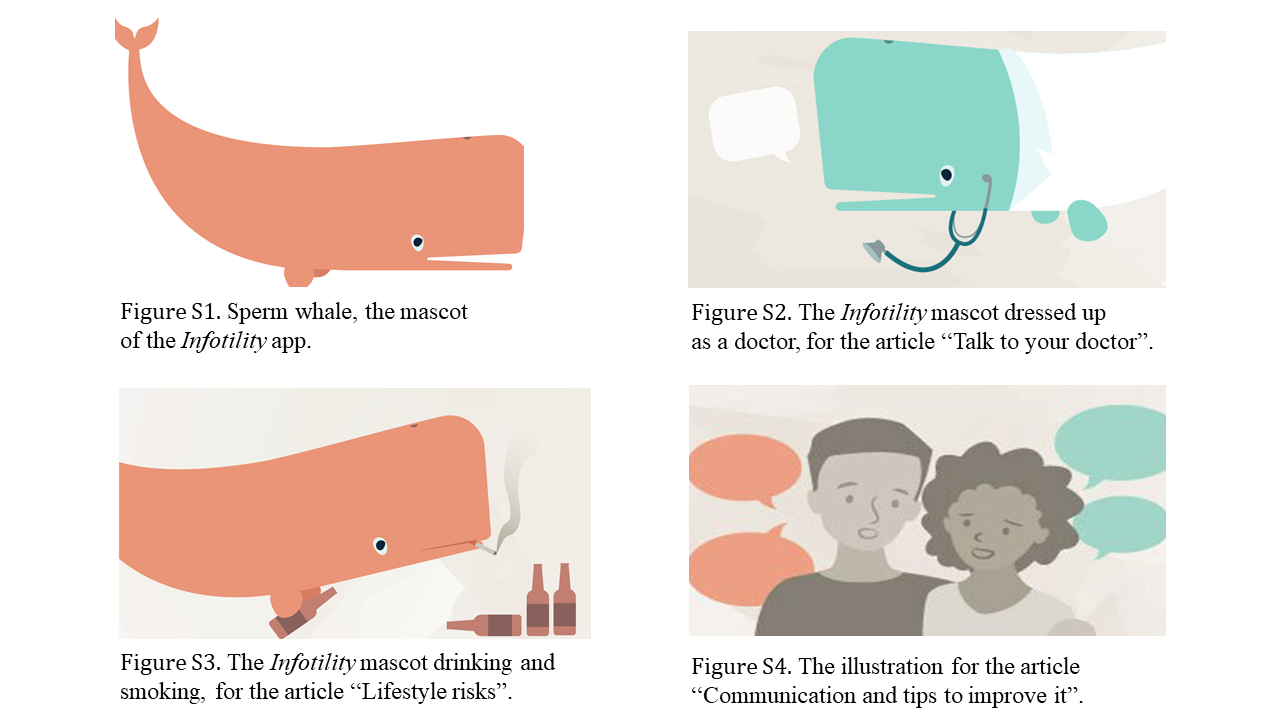

Supplement: Multimedia Appendix 3 [file formative_v5i10e28136_app3.png]
