# Supplementary material for: An mHealth App to Support Fertility Patients Navigating the World of Infertility (Infotility): Development and Usability Study
Source: JMIR Form Res. 2021 Oct 12;5(10):e28136. doi: 10.2196/28136 (PMC8548975; doi:10.2196/28136)
Supplement: Multimedia Appendix 4 [file formative_v5i10e28136_app4.pdf]

Socio-demographic characteristics of study participants (N=250)

	n	Valid % or *M*(*SD*)(Range)
**Age (years)**	248	35.5(4.8)(23-54)
**Gender**		
Male	65	26.0
Female	185	74.0
**Language**		
English	178	71.2
French	72	28.8
**How many children do you have?**		
None	194	78.5
1 or more	53	21.5
**Total household income (CAD)**		
≤ $40,000	14	5.7
$40,000-59,999	29	11.8
$60,000-79,999	26	10.6
$80,000-99,999	25	10.2
$100,000-119,999	44	18.0
$120,000-139,999	24	9.8
$140,000-159,999	23	9.4
$160,000 and above	60	24.5
**Highest level of education**		
Elementary	2	0.8
High school diploma	14	5.6
CEGEP[a], trade, vocational	46	18.5
University degree	79	31.9
Graduate or other professional degree	107	43.1
**Immigrant status**		
Immigrant	102	41.6
Born in Canada	143	58.4
**Ethnicity**		
White	151	61.1
Black	20	8.1
Latin, Central and South American	10	4.0
West Central Asian and Middle Eastern	22	8.9
South Asian	19	7.7
East and Southeast Asian	17	6.9
Mixed ethnicity	7	2.8

Other	1	0.4
**Do you consider yourself a religious person?**		
Yes	139	56.0
No	109	44.0

[a] CEGEP is a two-year college preparatory program or three-year technical program following high school and preceding post-secondary education in Quebec, Canada.
